# Coimmunoprecipitation with MYR1 Identifies Three Additional Proteins within the Toxoplasma gondii Parasitophorous Vacuole Required for Translocation of Dense Granule Effectors into Host Cells

**DOI:** 10.1128/mSphere.00858-19

**Published:** 2020-02-19

**Authors:** Alicja M. Cygan, Terence C. Theisen, Alma G. Mendoza, Nicole D. Marino, Michael W. Panas, John C. Boothroyd

**Affiliations:** a Department of Microbiology and Immunology, Stanford School of Medicine, Stanford, California, USA; University of Georgia

**Keywords:** *Toxoplasma*, immunoprecipitation, mass spectrometry, parasitology, protein export

## Abstract

Toxoplasma gondii is a ubiquitous, intracellular protozoan that extensively modifies infected host cells through secreted effector proteins. Many such effectors must be translocated across the parasitophorous vacuole (PV), in which the parasites replicate, ultimately ending up in the host cytosol or nucleus. This translocation has previously been shown to be dependent on five parasite proteins: MYR1, MYR2, MYR3, ROP17, and ASP5. We report here the identification of several MYR1-interacting and novel PV-localized proteins via affinity purification of MYR1, including TGGT1_211460 (dubbed MYR4), TGGT1_204340 (dubbed GRA54), and TGGT1_270320 (PPM3C). Further, we show that three of the MYR1-interacting proteins, GRA44, GRA45, and MYR4, are essential for the translocation of the *Toxoplasma* effector protein GRA16 and for the upregulation of human c-Myc and cyclin E1 in infected cells. GRA44 and GRA45 contain ASP5 processing motifs, but like MYR1, processing at these sites appears to be nonessential for their role in protein translocation. These results expand our understanding of the mechanism of effector translocation in *Toxoplasma* and indicate that the process is highly complex and dependent on at least eight discrete proteins.

**IMPORTANCE** *Toxoplasma* is an extremely successful intracellular parasite and important human pathogen. Upon infection of a new cell, *Toxoplasma* establishes a replicative vacuole and translocates parasite effectors across this vacuole to function from the host cytosol and nucleus. These effectors play a key role in parasite virulence. The work reported here newly identifies three parasite proteins that are necessary for protein translocation into the host cell. These results significantly increase our knowledge of the molecular players involved in protein translocation in *Toxoplasma*-infected cells and provide additional potential drug targets.

## Abstract (precis)

*Toxoplasma* is an extremely successful intracellular parasite and important human pathogen. Upon infection of a new cell, *Toxoplasma* establishes a replicative vacuole and translocates parasite effectors across this vacuole to function from the host cytosol and nucleus. These effectors play a key role in parasite virulence. The work reported here newly identifies three parasite proteins that are necessary for protein translocation into the host cell. These results significantly increase our knowledge of the molecular players involved in protein translocation in *Toxoplasma*-infected cells and provide additional potential drug targets.

## INTRODUCTION

Toxoplasma gondii is an obligate intracellular parasite that can cause severe illness in immunocompromised individuals and the developing fetus. It is estimated to infect up to a third of the world’s population and has an unparalleled host range, infecting virtually any nucleated cell in almost any warm-blooded animal (1). In order to survive within a host cell, *Toxoplasma* tachyzoites, the rapidly dividing, asexual stage of the parasite, establish a replicative niche, the parasitophorous vacuole (PV), whose membrane, the PV membrane (PVM), acts as the interface between parasite and host. While the PV protects intracellular *Toxoplasma* from clearance by the innate immune system, it also acts as a barrier that *Toxoplasma* must overcome in order to hijack host resources.

*Toxoplasma* extensively modifies the host cells that it infects via secreted effectors, either rhoptry (ROP) or dense granule (GRA) proteins, which it introduces into the host during or following invasion (2). In recent years, several *Toxoplasma* GRAs, including GRA16, GRA24, IST, HCE1/TEEGR, GRA28, and GRA18, that are translocated across the PVM into the host cell cytosol and/or nucleus, where they can have profound effects on host processes, have been identified (3–9). The machinery that is responsible for the translocation of these effectors across the *Toxoplasma* PVM is incompletely defined. A recent forward genetic screen identified several parasite proteins essential for GRA protein translocation, including MYR1, MYR2, and MYR3 (named for their effect on host c-Myc regulation) and the rhoptry-derived protein kinase, ROP17 (10–12). Precisely how these proteins function to promote protein translocation across the PVM is poorly understood. Of the four, the only protein with a known biochemical function is ROP17, a serine/threonine protein kinase that phosphorylates host and perhaps parasite proteins at the PVM (14).

In addition to MYR1, MYR2, MYR3, and ROP17, an active *Toxoplasma* aspartyl protease V (ASP5), which proteolytically processes secreted proteins at the amino acid sequence RRL (also known as a *Toxoplasma* export element [TEXEL]), is also required for the translocation of all exported GRAs studied thus far (5, 6, 8, 9, 15–17). In *Plasmodium*, the homolog of ASP5, plasmepsin V, appears to “license” many proteins for export across the PVM by proteolytically processing them at a *Plasmodium* export element (RXLXE/Q/D) (18–21). Intriguingly, and as for *Plasmodium* (22, 23), not all of *Toxoplasma*’s exported GRAs contain RRL motifs (e.g., GRA24, GRA28, and HCE1/TEEGR lack such an element), which leaves open the possibility that ASP5’s role in translocation is in processing the translocation machinery rather than the effectors themselves. Indeed, MYR1 is processed by ASP5, but this processing is not necessary for protein export, as unprocessed full-length MYR1 harboring a mutated RRL motif can still promote the translocation of the effector GRA24 to the host nucleus (24). The role of ASP5 processing of MYR1, therefore, remains unknown.

To learn more about the mechanism of protein translocation in *Toxoplasma* and to complement the genetic approaches taken previously, we report here on the use of MYR1 as bait for immunoprecipitation (IP) followed by mass spectrometry (MS) to identify putative MYR1-associated proteins that are involved in effector translocation. Of the many associating proteins, at least 11 are shown here or were previously known to be PV localized, and of these, 3 additional proteins are now shown to be required for GRA translocation across the PVM. Interestingly, all three of these new components contain RRL motifs, with two confirmed to be cleaved in an ASP5-dependent manner, yet, like MYR1, cleavage at these sites does not appear to be required for their translocation function. Thus, we have expanded the list of proteins involved in GRA translocation to eight while highlighting the question of why at least three of these components (MYR1, GRA44, and GRA45) are proteolytically processed without any apparent impact on their one known function.

## RESULTS

We previously reported the use of a forward genetic screen to identify *Toxoplasma* genes required for the induction of human c-Myc. This identified *MYR1*, *MYR2*, *MYR3*, and *ROP17* to be essential for the translocation of effector proteins across the PVM (10–12). Two of these proteins, MYR1 and MYR3, were found to coprecipitate with each other (11), and we hypothesized that MYR1 functions in complex with other yet unidentified proteins to facilitate effector translocation across the PVM. Given the small but significant reduction in plaque size observed when growing strains from which MYR1, MYR2, and MYR3 were deleted on human foreskin fibroblasts (HFFs) (11), we also reasoned that the genetic approach might also miss genes whose disruption substantially reduces fitness.

**FIG 1:**
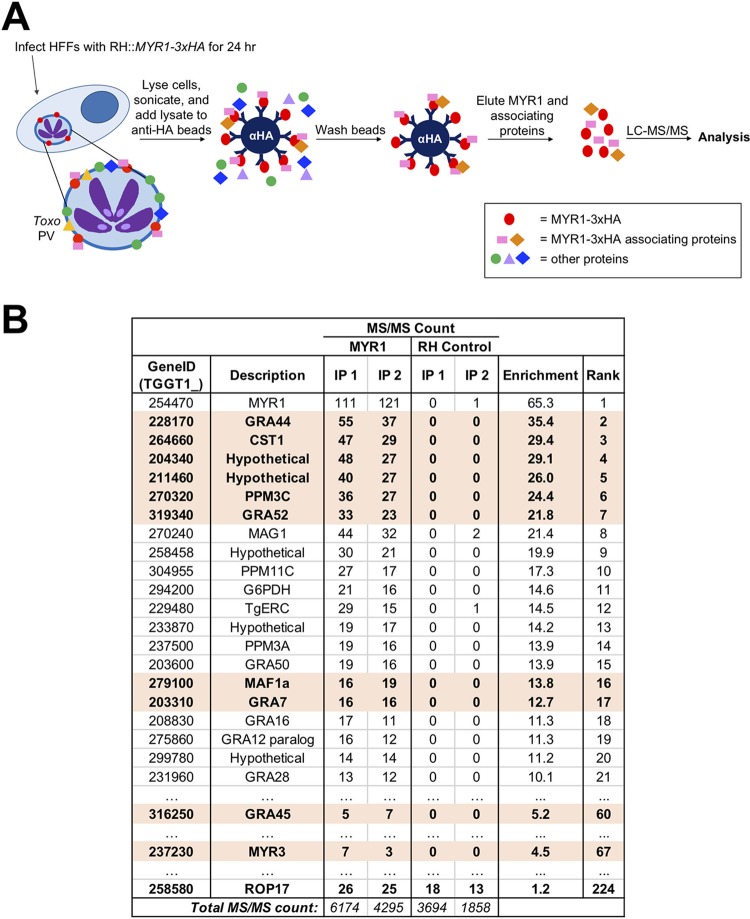
MYR1-3×HA immunoprecipitation identifies many MYR1-associating *Toxoplasma* proteins. (A) Schematic of MYR1 IP-MS work flow. (B) Results of IP-MS analysis. Mass spectrometry was performed on immunoprecipitated material as depicted in panel A, and the number of spectral counts was determined for all identified proteins. This experiment was performed twice (IP 1 and IP 2) for both RH::*MYR1-3×HA* and an RHΔ*hpt* untagged control. The *Toxoplasma* proteins identified from the two experiments were ranked according to the average NSAF enrichment in the MYR1-3×HA-expressing strain relative to that for the untagged RH control after adding a nominal single count to all results, enabling a ratio to be determined (Enrichment and Rank). The full data set, including associating host proteins, is presented in Data Set S1 in the supplemental material. Displayed here are the majority *Toxoplasma* protein identifiers (TGGT1_), i.e., the proteins that contained at least half of the peptides belonging to a group of proteins that could not be unambiguously identified by unique peptides; the descriptive name for each protein (Description); and the corresponding number of spectral counts detected (Total MS/MS count) for all *Toxoplasma* proteins with an average enrichment score of greater than 10. Also shown are data for the proteins GRA45, MYR3, and ROP17. The proteins whose genes chosen for subsequent disruption are highlighted in orange.

To identify additional MYR1-associating proteins, therefore, we adopted a biochemical approach. Specifically, we immunoprecipitated 3× hemagglutinin (3×HA)-tagged MYR1 from HFFs infected for 24 h with an RH::*MYR1-3×HA* strain or from an untagged RH strain to control for proteins that coprecipitate with the anti-HA beads nonspecifically (Fig. 1A). Liquid chromatography (LC)-tandem mass spectrometry (MS/MS) was performed on the eluates, and the identified parasite proteins were ranked by the ratio of the average normalized spectral abundance factors (NSAFs) for a given protein in the RH::*MYR1-3×HA* lysates compared to that in the RH control lysates (25). This mass spectrometry experiment was performed twice (IP 1 and IP 2). As expected, MYR1 was the most enriched protein in both biological replicates (Fig. 1B). Additionally, several PV- or PVM-localized GRA proteins were highly enriched (enrichment score > 10) in the MYR1-3×HA immunoprecipitations over that in the untagged RH control immunoprecipitations, including GRA44, CST1, GRA52, MAG1, PPM11C, GRA50, MAF1a, GRA7, and a GRA12 paralog, in addition to two exported effector proteins, GRA16 and GRA28, of which GRA16 has been shown to be exported in a MYR1-dependent manner (10) (Fig. 1B; see also Data Set S1 in the supplemental material). While CST1 has traditionally been thought to be specific to the slow-growing bradyzoite stage of *Toxoplasma*, due to its detection in the cyst wall (26, 27), our results, along with those of others (28), provide clear evidence for CST1 protein expression in tachyzoites. The large number of enriched PV- and PVM-localized proteins may be explained by the mild detergent conditions used (0.1% NP-40), which were chosen in an attempt to maintain associating proteins, although these proteins might also be associating with one another in large, nonspecific complexes or lipid rafts (29).

10.1128/mSphere.00858-19.7 DATA SET S1 Mass spectrometry analysis parameters and results for proteins that coimmunoprecipitate with MYR1-3×HA-expressing and untagged RH parasites. Shown for all sheets are the identifiers corresponding to the majority proteins, i.e., the proteins which contained at least half of the peptides belonging to a protein group (grouping of proteins which cannot be unambiguously identified by unique peptides); the number of spectral counts (MS/MS count); the average NSAF enrichment score (MYR1/RH Enrichment, as further elaborated in Materials and Methods); and the protein rank, as defined by the enrichment score corresponding to each grouping. The gene product (for *Toxoplasma* proteins) or associated gene name (for human proteins) for the first listed protein identifier in each row is shown in the Description column. Sheet 1 (Toxo_proteins) shows the experimental data sets for *Toxoplasma* proteins only, listed in rank order by the average NSAF enrichment from both experiments. Sheet 2 (All_proteins) shows the experimental data sets for both human and *Toxoplasma* proteins, listed in rank order by the average NSAF enrichment from both experiments. Sheet 3 (Parameters) shows the parameters used in the MaxQuant analysis. Download Data Set S1, XLSX file, 0.10 MB. Copyright © 2020 Cygan et al. 2020 Cygan et al. This content is distributed under the terms of the Creative Commons Attribution 4.0 International license.

Importantly, and also as expected, the known MYR1-associating protein, MYR3, was enriched in the MYR1-3×HA immunoprecipitations, albeit with an enrichment score (4.5) that did not put it in the top 20 most enriched proteins (Fig. 1B; Data Set S1). Of note, ROP17 was not substantially enriched (enrichment score = 1.2), and no peptides for MYR2 were detected, but neither protein has previously been found to associate with MYR1, and so this was not unexpected. Human proteins with an enrichment score of >10 include Filamin-C (FLNC), DNA-dependent protein kinase catalytic subunit (PRKDC), sarcoplasmic/endoplasmic reticulum calcium ATPase 2 (ATP2A2), and alpha-*N*-acetylglucosaminidase (NAGLU) (Data Set S1). As our focus was on parasite proteins only, the potential role of these human proteins in *Toxoplasma* infection was not further investigated.

To screen for a possible role in GRA effector translocation, we focused on the top 6 most enriched parasite proteins: GRA44 (TGGT1_228170), CST1 (TGGT1_264660), TGGT1_204340 (here abbreviated 204340), TGGT1_211460 (here abbreviated 211460), PPM3C (TGGT1_270320), and GRA52 (TGGT1_319340). GRA45 (TGGT1_316250) was also pursued because it is a known binding partner of the top hit, GRA44 (30), and it also had a substantial enrichment score of 5.2 in the immunoprecipitations (Fig. 1B). Interestingly, two well-characterized PV proteins that have not previously been described to be involved in effector translocation, GRA7 (TGGT1_203310) and MAF1a (TGGT1_279100), were substantially enriched, and since antibodies and gene knockouts for both were readily available, we included these in the list of genes to explore further. Lastly, as a positive control for a protein whose disruption is known to prevent effector translocation, we also included MYR3 in the pipeline for gene disruption and testing. All 10 proteins chosen for further analysis are highlighted in orange in Fig. 1B.

**FIG 2:**
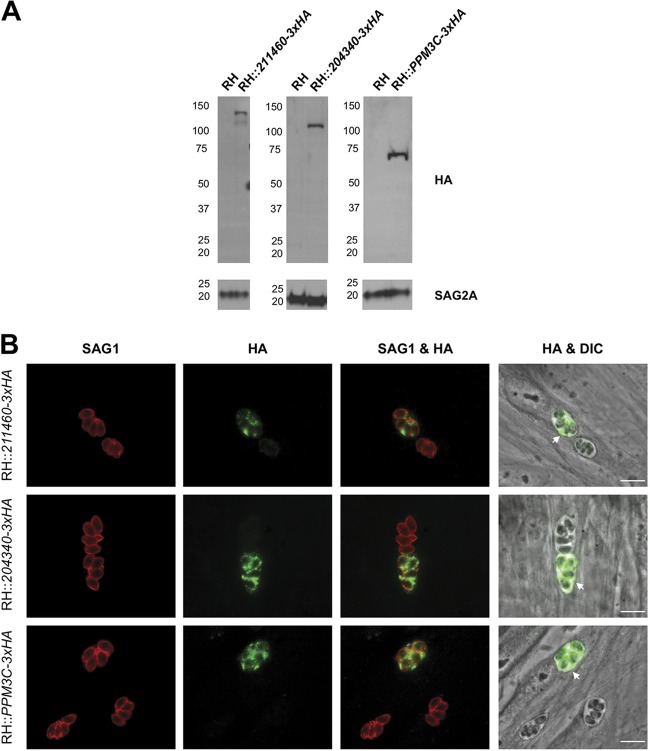
211460, 204340, and PPM3C localize to the *Toxoplasma* parasitophorous vacuole in infected cells. (A) Western blot of endogenously tagged parasite proteins. HFFs were infected with RHΔ*hpt*Δ*ku80* tachyzoites (RH) or with populations of RH that had been transfected with HA-tagged plasmids targeted to the indicated locus (RH::*211460-3×HA*, RH::*204340-3×HA*, and RH::*PPM3C-3×HA*). Lysates from infected HFFs were prepared, and the HA-tagged proteins were detected by Western blotting and probing with rat anti-HA antibodies. Rabbit anti-SAG2A staining was used as a loading control for total parasite protein. The approximate migration of a ladder of size standards (indicated in kilodaltons on the left) is indicated. (B) Representative immunofluorescence microscopy images of endogenously tagged parasite proteins. The populations of endogenously tagged parasites analyzed in the assay whose results are presented in panel A were allowed to infect HFFs for 16 h before the infected monolayers were fixed with methanol. The corresponding tagged proteins in parasites that had successfully incorporated the HA tag were detected with rat anti-HA antibodies, while all tachyzoites were detected with mouse anti-SAG1 antibodies, and the entire monolayer was visualized by differential interference contrast (DIC) microscopy. The arrows indicate the localization of the endogenously tagged proteins outside of the parasites and within the PV. Bars = 10 μm.

With the exception of ASP5, and as might be expected, all proteins so far published to be required for effector translocation across the PVM localize to the PV/PVM (2). Of the 10 proteins chosen for further analysis, GRA44, GRA45, CST1, GRA7, GRA52, and MAF1a are all known to be PV/PVM localized (26, 30–37). The localization of 211460 and PPM3C has not been reported, but both include predicted signal peptides (see below), as does 204340, which has been described to possibly be micronemal (34). We therefore set out to localize these three proteins within infected cells. To do this, we generated populations of parasites in which each of the three genes was endogenously modified to encode a 3×HA tag immediately before the stop codon and then assessed the protein’s localization by immunofluorescence assay (IFA). Correct integration of the 3×HA tag into the appropriate locus was confirmed by PCR and by checking for an appropriately sized HA-tagged protein via Western blotting. The results (Fig. 2A) showed major bands at ∼130 kDa, ∼110 kDa, and ∼70 kDa for 211460, 204340, and PPM3C, respectively. In the case of 204340 and PPM3C, these are close to the predicted sizes of ∼97 kDa and ∼60kDa, respectively (ToxoDB Toxoplasma gondii genome database, v45). For 211460, however, the mobility was significantly retarded relative to its predicted size of ∼100 kDa. This could be due to its acidic pI of 4.91 (ToxoDB, v45), which is known to reduce protein mobility on SDS-PAGE (35), and/or to posttranslational modifications (all three proteins are reported to potentially be phosphorylated, with one predicted phosphorylation site for MYR4, one for PPM3C, and nine for GRA54 [52] [ToxoDB, v45]). This same slower-than-expected mobility for the major band was seen for an independently generated, cloned line expressing HA-tagged 211460 (Fig. S1A), and so we conclude that this is the correct mobility for this protein. Interestingly, both the 3×HA-tagged 211460 population and the single clone also showed a smaller but considerably weaker band at about the expected size (∼100 kDa). Whether this smaller 211460 product is biologically relevant or is simply a product of protein degradation is unclear.

10.1128/mSphere.00858-19.1 FIG S1 (A) Western blot of endogenously tagged 211460-3×HA single clone and population. HFFs were infected with RHΔ*hpt*Δ*ku80* tachyzoites (RH) or endogenously tagged RH::*211460-3×HA* parasites (from either the population or an independently generated single clone). Lysates from infected HFFs were prepared, and 211460-3×HA was detected by Western blotting using rat anti-HA antibodies. Rabbit anti-SAG2A staining was used as a loading control for total parasite protein. The Western blot for the 211460-3×HA population presents the same data presented in Fig. 2A. The approximate migration of a ladder of size standards (indicated in kilodaltons) is indicated. (B) Immunofluorescence microscopy of endogenously tagged 211460-3×HA from an independently generated single clone. Tachyzoites were allowed to infect HFFs for 16 h before the infected monolayer was fixed with methanol. 211460-3×HA was detected with rat anti-HA antibodies, *Toxoplasma* tachyzoites were detected with mouse anti-SAG1 antibodies, and the infected monolayer was visualized by differential interference contrast (DIC) microscopy. Bars = 10 μm. Download FIG S1, PDF file, 0.6 MB. Copyright © 2020 Cygan et al. 2020 Cygan et al. This content is distributed under the terms of the Creative Commons Attribution 4.0 International license.

Using the HA-tagged 211460, 204340, and PPM3C parasite populations, we next sought to determine the localization of these proteins in infected cells. As determined using SignalP software (v5.0), all three proteins had strongly predicted signal peptides, although in the case of 211460, this was true only if translation starts at the fourth in-frame methionine (position 61) relative to the protein sequence predicted on ToxoDB (v45). The results (Fig. 2B) show a clear PV-like signal outside of the parasites in the 211460-3×HA, 204340-4×HA, and PPM3C-3×HA populations, including at the periphery of the PV. The PV localization for 211460 was further confirmed in the independently generated clonal line (Fig. S1B). Thus, we conclude that 211460, 204340, and PPM3C are at least transiently localized to the *Toxoplasma* PV during infection. Furthermore, we also assessed the localization of these proteins within the parasites themselves. The results (Fig. S2) showed that while PPM3C appears to be present throughout the parasite, 211460 and 204340 show a clear, punctate staining pattern that largely colocalizes with the dense granule protein GRA7, suggesting that these two proteins are also GRA proteins. We therefore designate *204340* as *GRA54* for its GRA-like localization and *211460* as *MYR4* for the reasons described below.

10.1128/mSphere.00858-19.2 FIG S2 Immunofluorescence microscopy of endogenously tagged proteins in extracellular parasites. The populations of endogenously tagged parasites that were analyzed and for which the results are shown in Fig. 2A were seeded onto empty coverslips before being fixed with methanol. The corresponding tagged proteins were detected with rat anti-HA antibodies; the marker for dense granule proteins, GRA7, was detected with rabbit anti-GRA7 antibodies; and the parasites were visualized with differential interference contrast (DIC) microscopy. Bars = 5 μm. Download FIG S2, PDF file, 0.6 MB. Copyright © 2020 Cygan et al. 2020 Cygan et al. This content is distributed under the terms of the Creative Commons Attribution 4.0 International license.

**FIG 3:**
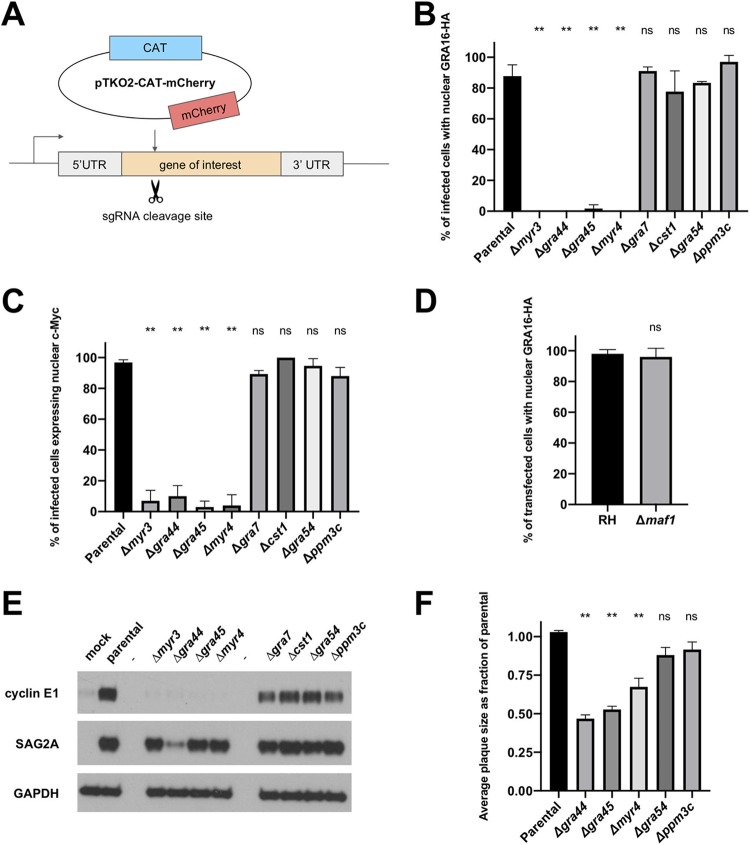
GRA44, GRA45, and MYR4 are required for *Toxoplasma* effector translocation and fully efficient growth *in vitro*. (A) Schematic of CRISPR-mediated gene disruption of candidate genes, followed by insertion of the pTKO2 plasmid carrying the gene for mCherry and a chloramphenicol acetyltransferase (CAT) gene for selection in chloramphenicol. UTR, untranslated region. (B) Quantification of the percentage of infected cells showing GRA16-HA in the host nucleus via IFA. Tachyzoites were allowed to infect HFFs for 16 h before the infected monolayers were fixed with methanol and stained with rat anti-HA antibodies. The averages are based on examination of at least 25 infected host cells per experiment from 2 to 5 biological replicates, and error bars indicate the standard deviation (SD). Statistics were performed using one-way analysis of variance and Dunnett’s multiple-comparison test. *P* values are for the indicated strain relative to the parental control. **, *P* < 0.0001; ns, not significant (*P* > 0.05). (C) Quantification of the percentage of infected cells showing the upregulation of the human c-Myc protein in the host nucleus via IFA. Tachyzoites were allowed to infect HFFs in serum-free medium for 20 h before the infected monolayers were fixed with methanol and stained with rabbit anti-c-Myc antibodies. Scoring and statistics are as described in the legend to panel B. (D) Quantification of the percentage of transfected, infected cells showing GRA16-HA in the host nucleus via IFA. Wild-type RHΔ*hpt* and RHΔ*maf1* tachyzoites were transiently transfected with a plasmid expressing GRA16-HA, and transfected parasites were allowed to infect HFFs for 16 h before the infected monolayers were fixed with methanol and stained with rat anti-HA antibodies. The averages are based on the examination of 25 vacuoles from 2 biological replicates, and error bars indicate the SD. Statistics are as described in the legend to panel B. (E) Western blot of human cyclin E1 protein in infected cells. HFFs were infected with the indicated tachyzoites or mock treated with uninfected HFF lysate for 18 h before lysates were generated for immunoblotting. Lysates were analyzed by Western blotting using mouse anti-cyclin E1 antibodies. Rabbit anti-SAG2A and mouse anti-GAPDH were used to assess the levels of parasite and host proteins in the lysate, respectively. Lanes −, empty lanes. (F) Quantification of plaque size. HFFs were infected with tachyzoites of the indicated strain for 7 days, fixed with methanol, and then stained with crystal violet. Plaque size was measured using ImageJ software. Plaque areas were normalized to the median for the parental strain for each biological replicate. The averages are based on the results of at least 3 independent biological replicates, each with 2 to 3 technical replicates, and error bars represent the standard error of the mean. Statistics are as described in the legend to panel B.

To assess their potential involvement in GRA effector translocation, we attempted to generate knockouts of our candidate genes in a strain of *Toxoplasma* that constitutively expresses an HA-tagged version of the MYR1-dependent secreted effector protein GRA16, RHΔ*gra16*::*GRA16-HA* (the parental strain). To do this, we cotransfected a CRISPR/Cas9 single guide RNA (sgRNA) plasmid that targets the first exon of the relevant gene along with a pTKO2-CAT-mCherry plasmid (CAT is chloramphenicol acetyltransferase and is encoded by the chloramphenicol resistance gene; Fig. 3A). Following selection with chloramphenicol, we cloned the populations by limiting dilution and confirmed disruptive integration of the vector by PCR with gene-specific primers. Using this strategy, we were able to disrupt the genomic loci of *MYR3*, *GRA44*, *GRA45*, *CST1*, *GRA54*, *MYR4*, *PPM3C*, and *GRA7* (Fig. S3). Despite several attempts, however, we were unable to generate a *GRA52* mutant. This gene may be essential, as it has a very negative CRISPR fitness score of −3.96 (36). Given that the *MAF1* locus is expanded in *Toxoplasma*, with 4 copies in RH parasites (37), we chose not to attempt a CRISPR/Cas9 approach to knock out MAF1a and instead utilized a previously generated strain in which the entire *MAF1* cluster (including the regions encoding MAF1a and MAF1b) was deleted (33).

10.1128/mSphere.00858-19.3 FIG S3 Schematic of CRISPR-mediated gene disruption of candidate genes. Primers flanking the guide-targeted region, indicated by Forward and Reverse, were constructed to amplify an ∼1,000-bp region of the native, uninterrupted gene. pTKO2-CAT-mCherry was the plasmid used for integration and selection. (A) PCR amplifications of genomic DNA from RHΔ*gra16*::GRA16-HA parasites (parental) and from a chloramphenicol-resistant (CAT^+^) clonal strain with disruption of the indicated gene, using the forward and reverse primers shown in panel A. The sizes (in base pairs) of the standard ladder are shown. Bands of the expected size in the parental strain (∼1,000 bp) and either the lack of a band or the presence of altered bands in the disrupted strains indicate insertion of the selection plasmid within the targeted gene, as indicated (e.g., Δ*myr3* is a strain with a disruption of the *MYR3* locus). Download FIG S3, PDF file, 0.5 MB. Copyright © 2020 Cygan et al. 2020 Cygan et al. This content is distributed under the terms of the Creative Commons Attribution 4.0 International license.

To determine if the absence of any of the candidate genes results in a defect in effector translocation across the PVM, we used IFA to assess both GRA16-HA export to the host nucleus and host c-Myc upregulation (which *Toxoplasma* induces during infection [38]) in the disrupted lines. Quantified results for all nine genes tested showed that disruption of *GRA44*, *GRA45*, *MYR4*, and the previously described *MYR3* all resulted in a complete or nearly complete block in GRA16 export to the host nucleus (Fig. 3B; Fig. S4) and a failure to upregulate host c-Myc (Fig. 3C; Fig. S4); on the other hand, disruption of *GRA7*, *CST1*, *GRA54*, or *PPM3C* resulted in no detectable effect on either of these two phenotypes. Additionally, we found that the previously generated *Δmaf1* strain also had normal GRA16 export to the host nucleus (Fig. 3D). These results indicate that of the nine genes tested here, only *MYR3*, *GRA44*, *GRA45*, and *MYR4* are necessary for the translocation of GRA effectors across the PVM.

10.1128/mSphere.00858-19.4 FIG S4 Immunofluorescence microscopy of GRA16-HA nuclear localization and human nuclear c-Myc expression in HFFs infected with the indicated disrupted parasite strains. Tachyzoites were allowed to infect HFFs (without serum) for 18 h before the infected monolayers were fixed with methanol and stained with rat anti-HA antibodies and rabbit anti-c-Myc antibodies. Host nuclei were visualized using DAPI. Bars = 20 μm. Download FIG S4, PDF file, 2.9 MB. Copyright © 2020 Cygan et al. 2020 Cygan et al. This content is distributed under the terms of the Creative Commons Attribution 4.0 International license.

To test the generality of their role in effector translocation, we next assessed the impact of these gene disruptions on the upregulation of host cyclin E1, which has been shown to be dependent on export of the MYR1-dependent effector HCE1/TEEGR (6). The results showed that, as for GRA16, the disruption of *MYR3*, *GRA44*, *GRA45*, and *MYR4* also resulted in a block in cyclin E1 upregulation in infected host cells, while no obvious defect was observed in the parasite lines disrupted in *GRA7*, *CST1*, *GRA54*, and *PPM3C* (Fig. 3E). A repetition of the cyclin E1 Western blot assay with a higher parasite input revealed that the absence of cyclin E1 upregulation observed in *Δgra44* parasites in Fig. 3E was not due to a low parasite input in that particular experiment (Fig. S5). These results argue that *GRA44*, *GRA45*, and *MYR4* are all required for translocation across the PVM of at least two independent GRA effectors.

10.1128/mSphere.00858-19.5 FIG S5 Western blot of human cyclin E1 protein in cells infected with the indicated parasite strain. HFFs were infected with the indicated strain of tachyzoites or mock treated with uninfected HFF lysate for 20 h before lysates were generated for immunoblotting. Lysates were analyzed by Western blotting using mouse anti-cyclin E1 antibodies. Rabbit anti-SAG2A was used to assess the levels of parasite protein in the lysate. Download FIG S5, PDF file, 0.04 MB. Copyright © 2020 Cygan et al. 2020 Cygan et al. This content is distributed under the terms of the Creative Commons Attribution 4.0 International license.

Our previous work has shown that the deletion of *MYR1*, *MYR2*, and *MYR3* results in a small but significant, negative effect on parasite growth *in vitro* (11). To determine if disruption of the three new genes involved in effector translocation described here has a similar impact, we infected HFF monolayers with each of the disrupted lines, fixed the monolayers at 7 days postinfection, and measured the plaque size. The results showed that the *Δmyr4*, *Δgra44*, and *Δgra45* strains all exhibited a significant growth defect compared to the parental strain (Fig. 3F). The *Δgra54* and *Δppm3c* strains, on the other hand, did not have significant growth defects, consistent with the growth defects observed being dependent on the respective genotype rather than nonspecific effects of the manipulations.

**FIG 4:**
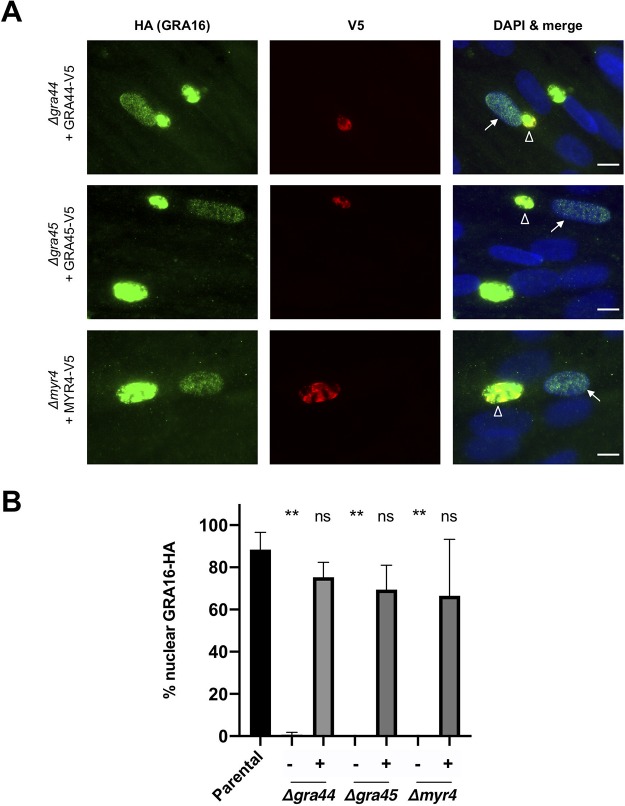
Ectopic protein expression rescues the effector translocation defect in Δ*gra44*, Δ*gra45*, and Δ*myr4* parasites. (A) Representative immunofluorescence microscopy images of transiently expressed GRA44, GRA45, and MYR4 proteins. The indicated strains were transiently transfected with plasmids expressing the corresponding, C-terminally V5-tagged protein under the control of its native promoter, and the tachyzoites were allowed to infect HFFs for 18 to 22 h before the infected monolayers were fixed with methanol. Localization of the V5-tagged proteins and rescue of the GRA16-HA host nuclear translocation were assessed by IFA using mouse anti-V5 and rat anti-HA antibodies, respectively. White arrows indicate a GRA16-HA-positive host nucleus in a cell infected with tachyzoites expressing the indicated V5 tagged protein (white open arrowheads). Bars = 10 μm. (B) Quantification of the data represented in panel A, showing the percentage of infected cells showing GRA16-HA in the host nucleus via IFA. The indicated strains were transiently transfected with either an empty plasmid (−) or plasmids expressing the corresponding C-terminally V5-tagged protein (+) under the control of its native promoter. Scoring and statistics are as described in the legend to Fig. 3B, except for the conditions with C-terminally V5-tagged protein (+), where only cells infected with V5-positive vacuoles were quantified.

To confirm that ablation of the *GRA44*, *GRA45*, and *MYR4* loci is responsible for the observed defect in GRA16 export, we transiently expressed a C-terminally V5-tagged version of each protein, driven by its native promoter, in the relevant disrupted line. These transiently transfected parasites were then assessed for GRA16-HA export to the host nucleus via IFA. The results showed that the parental and complemented strains had GRA16-HA signals in both the vacuole and the host nucleus, while the parasites within the population that did not express the complementing transgene (as indicated by a lack of anti-V5 staining) showed essentially no GRA16 in the host nucleus (Fig. 4A and B). Thus, *GRA44*, *GRA45*, and *MYR4* are indeed essential for the translocation of effectors across the PVM, and we therefore designate *211460* as *MYR4*, consistent with previous nomenclature (10, 11).

**FIG 5:**
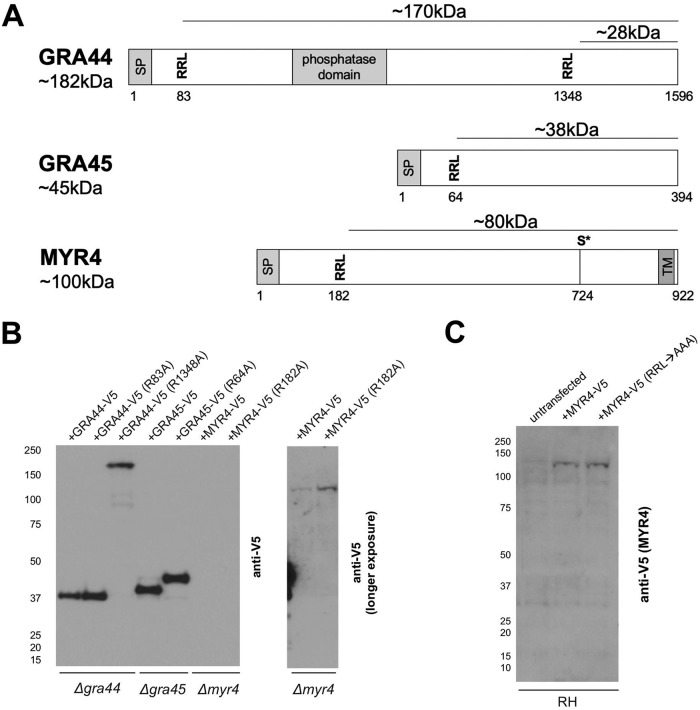
GRA44 and GRA45, but not MYR4, show evidence for processing at RRL sites. (A) Schematic of GRA44, GRA45, and MYR4 protein sequences, showing the locations of predicted signal peptides (SP), RRL tripeptide sequences, previously identified phosphorylated serine residues (S*) (52), and conserved domains, numbered in amino acid residues relative to the predicted N terminus of the primary translation product. The approximate molecular weights (in kilodaltons) of the indicated portions are indicated. The amino acid sequence of MYR4 was determined using the fourth in-frame methionine relative to the protein predicted in ToxoDB (v45). Transmembrane domain prediction was based on Phobius (51). (B) Western blot of protein processing. (Left) The indicated parasite lines were transiently transfected with plasmids expressing C-terminally V5-tagged versions of either the indicated wild-type protein or a mutant version with the indicated RRL mutated to ARL (the numbers at the top indicate the amino acid position of the mutated arginine). These were then used to infect HFFs for 18 h. Lysates were analyzed by Western blotting using mouse anti-V5 antibodies to detect the C-terminally V5-tagged portions of each protein. The approximate migration of a ladder of size standards (indicated in kilodaltons on the left) is indicated. (Right) A longer exposure of the right-most two lanes of the left panel. (C) Western blot of MYR4 processing. RHΔ*hpt* (RH) parasites were transiently transfected with either the wild type or an RRL → AAA-mutated version of C-terminally V5-tagged MYR4 and allowed to infect HFFs for 24 h before lysates were generated for immunoblotting. Lysates were analyzed by Western blotting using mouse anti-V5 antibodies to detect MYR4. The approximate migration of a ladder of size standards (indicated in kilodaltons on the left) is indicated.

Interestingly, GRA44, GRA45, and MYR4 all contain one or two instances of the 3-amino-acid motif RRL (Fig. 5A), which has previously been shown to be the preferred sequence for cleavage by ASP5 protease (15). Indeed, ASP5 cleavage at the sites shown in GRA44 and GRA45 (30), as well as at the first RRL site in the secreted GRA effector, GRA16 (15), has been experimentally confirmed. ASP5 is essential for the translocation of all GRA effectors so far tested (5, 6, 8, 9, 15–17), and it has previously been suggested that ASP5-mediated cleavage of some effectors is required to “license” them for translocation across the PVM, as appears to be the case in *Plasmodium* (18, 19). Given, however, that not all such effectors contain ASP5 processing motifs (e.g., GRA24 lacks the canonical RRL and shows no evidence of ASP5-dependent processing [17]) and given that the three newly identified components of the translocation machinery identified here do, we hypothesized that ASP5’s essential contribution to effector translocation across the PVM might be in processing one or more components of the translocation machinery. We have previously shown that MYR1 is also processed by ASP5 at an RRL site, but this does not appear to be required for MYR1 to function in effector translocation (24), and so we turned our attention to the newly identified translocation components identified here.

To determine if processing at the RRL sites of GRA44, GRA45, and MYR4 is required for protein translocation activity, we mutated the ASP5 cleavage sites by converting the first arginine to an alanine (i.e., RRL → ARL) in the V5-tagged complementation plasmids for each gene and transiently transfected these into the corresponding disrupted line. Western blots were then used to show that processing of GRA45 at its lone RRL and of GRA44 at its second RRL is indeed abrogated by the mutations (Fig. 5B). For the more N-terminal site in GRA44 (R83A), we cannot definitively confirm that the mutation abrogates ASP5 processing because GRA44 is epitope tagged at its C terminus, and so, assuming that cleavage at the two sites is an independent event, cleavage at the downstream site produces a C-terminal, V5-tagged fragment whether or not cleavage occurs at R83A. We fully expect, however, that the RRL → ARL change disrupts ASP5 cleavage at this site because it did in the two other examples shown here (GRA45 and the downstream site in GRA44, R1348A) and because RRL → ARL single point mutations have previously been shown to disrupt the ASP5-dependent cleavage of other proteins (24, 30). Furthermore, previous work to detect ASP5-dependent cleavage peptides using an N-terminal amine isotopic labeling of substrates (N-TAILS) approach on naturally occurring tachyzoite proteins identified peptides generated by cleavage immediately after this first RRL site in GRA44 (30); thus, this site is a site of natural cleavage and very likely to be a bona fide ASP5 processing site.

Interestingly, mutation of RRL to ARL in MYR4 did not appear to affect processing of the protein (Fig. 5B). To rule out whether this is due to incomplete ablation of the potential ASP5 processing site with just a single amino acid substitution, we assessed the processing of an RRL → AAA MYR4 mutant where the entire ASP5 processing motif was mutated to alanines. The results (Fig. 5C) showed that the higher-molecular-weight product of MYR4 (∼130 kDa) did not change in mobility upon mutation of the entire RRL motif, and thus, we conclude that little, if any, MYR4 is processed by ASP5. Note that, despite repeated attempts with large amounts of DNA, the signal for the transiently expressed MYR4 was never strong enough to confidently conclude whether a small amount of a processed form might be present in these transiently transfected parasites; we therefore cannot comment on whether the low-intensity, lower-molecular-weight product of MYR4 (∼100 kDa) seen in long exposures of endogenously tagged wild-type MYR4 (Fig. 2A; Fig. S1A) is a result of an ASP5 processing event.

**FIG 6:**
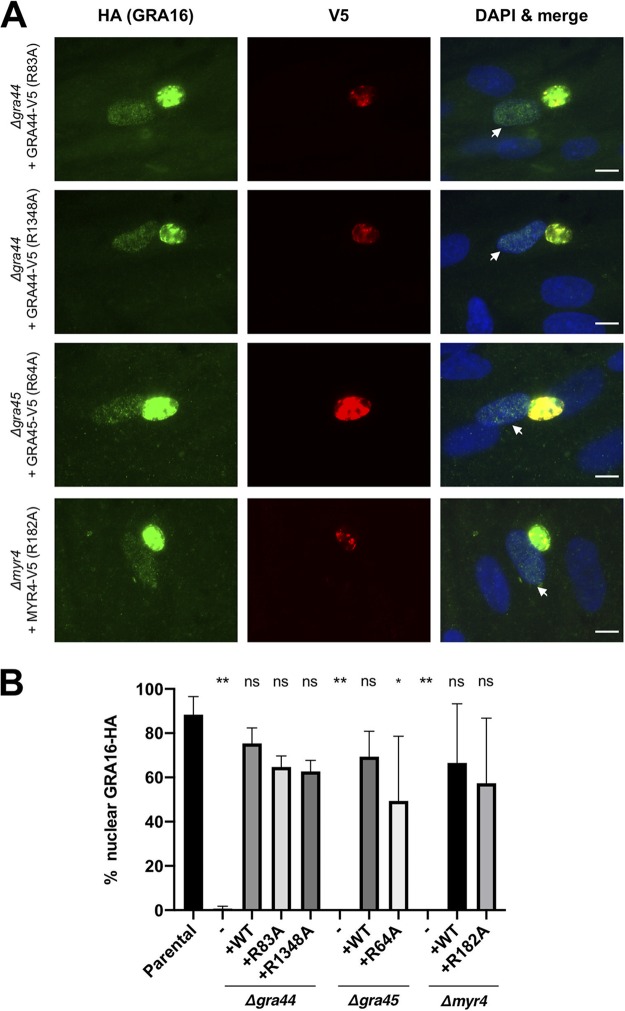
Ectopic expression of RRL mutants of GRA44, GRA45, and MYR4 rescues the translocation defect in Δ*gra44*, Δ*gra45*, and Δ*myr4* parasites. (A) Representative immunofluorescence microscopy images of transiently expressed GRA44, GRA45, and MYR4 RRL → ARL-mutated proteins. The indicated strains were transiently transfected with plasmids expressing the corresponding C-terminally V5-tagged protein under the control of its native promoter, and the tachyzoites were allowed to infect HFFs for 18 to 22 h before the infected monolayers were fixed with methanol. The localization of the V5-tagged proteins and rescue of the GRA16-HA host nuclear translocation were assessed by IFA using mouse anti-V5 and rat anti-HA antibodies, respectively. White arrows indicate a GRA16-HA-positive host nucleus in a cell infected with tachyzoites expressing the indicated V5-tagged protein. Bars = 10 μm. (B) Quantification of the data represented in panel A, showing the percentage of infected cells showing GRA16-HA in the host nucleus via IFA. The indicated strains were transiently transfected with either an empty plasmid (−) or plasmids expressing the corresponding C-terminally V5-tagged protein (+) under the control of its native promoter. The data for the untransfected parental strain, the empty plasmid-transfected strains, and the wild-type protein-transfected strains are the same as those in Fig. 4B and are included here for ease of comparison. Scoring and statistics are as described in the legend to Fig. 3B, except that the single asterisk indicates a *P* value of 0.017.

Having generated the four RRL → ARL mutants and having validated that ASP5 cleavage is ablated in at least two instances, we next tested each for its impact on the localization of the epitope-tagged, C-terminal portion of the protein and on the ability of the uncleaved protein to function, i.e., whether it can rescue the defect in effector protein translocation. The results showed that the RRL → ARL-mutated versions of each protein were still secreted into the PV, similar to the findings for the wild-type copy (Fig. 4A and 6A), and all were able to rescue the translocation defect to an extent similar to that for the corresponding control (wild-type) plasmid (Fig. 6B). While the GRA45 R64A mutant did substantially rescue translocation, it did not consistently rescue it to wild-type levels. Nevertheless, these data suggest that mutation of the RRL sites in GRA44, GRA45, and MYR4 to ARL does not substantially affect their function in effector protein translocation.

## DISCUSSION

Using affinity purification of MYR1 under conditions expected to retain associating partners, we identified three novel parasite proteins, GRA44, GRA45, and MYR4, to be essential for the export of GRA effectors into infected cells. Additionally, we localized MYR4, as well as two additional MYR1-associating proteins, GRA54 and PPM3C, to the PV in infected cells. Altogether, eight proteins are now known to be necessary for effector export: the three described here and MYR1, MYR2, MYR3, ROP17, and ASP5, summarized in Table S1 in the supplemental material (10–12, 15–17). Besides ASP5, which localizes to the Golgi apparatus (15–17), these proteins all localize to the PV/PVM.

10.1128/mSphere.00858-19.6 TABLE S1 Summary of *Toxoplasma* genes necessary for effector translocation. The number of predicted transmembrane domains, the number of RRL motifs, and the CRISPR phenotype score are listed for each *Toxoplasma* gene necessary for effector translocation identified thus far. Additionally, the percent identities of each of these genes to their orthologs in Hammondia hammondi and Neospora caninum and whether the RRL sequences are conserved in these species are also listed. Transmembrane domain prediction is based on Phobius [L. Kall, A. Krogh, and E. L. L. Sonnhammer, Nucleic Acids Res 35(Suppl 2):W429–W432, 2007, https://doi.org/10.1093/nar/gkm256]. CRISPR phenotype scores are from Sidik et al. (S. M. Sidik, D. Huet, S. M. Ganesan, M. H. Huynh, et al., Cell 166:1423–1435.e12, 2016, https://doi.org/10.1016/j.cell.2016.08.019). Identity was calculated by comparison to head-to-head comparison of the ortholog in the indicated species using the Sequence Manipulation Suite (P. Stothard, Biotechniques 28:1102–1104, 2018, https://doi.org/10.2144/00286ir01). Download Table S1, DOCX file, 0.01 MB. Copyright © 2020 Cygan et al. 2020 Cygan et al. This content is distributed under the terms of the Creative Commons Attribution 4.0 International license.

The newly identified components described here do not display any homology to known protein translocation machinery, based on BLAST analysis results (BLASTP, v2.10.0+), making it difficult to infer their functions, and thus, which, if any, are part of an actual translocon remains unknown. In addition to lacking homology to known translocation machinery, MYR4 and GRA45 do not have detectable homology to any other known, functional protein domains, nor do they share homology to proteins in any species outside of *Coccidia/Eimeriorina*. Like MYR1, MYR2, MYR3, and ROP17, however, MYR4, GRA44, and GRA45 all have clear orthologs in Hammondia hammondi and Neospora caninum (Table S1).

GRA44, in contrast, contains a putative phosphatase domain that shares homology to a region of the *Plasmodium* serine/threonine phosphatase UIS2 (28% identity over 21% of the protein; BLASTP, v2.10.0+), which has recently been shown to localize to the *Plasmodium* PVM in liver-stage parasites (39). Whether UIS2 plays a role in protein translocation in *Plasmodium* remains to be determined, but it would be surprising if it did, given that none of the other components of the complex known to promote translocation in *Plasmodium* (known as PTEX) so far studied play a role in translocation in *Toxoplasma* (2). Additionally, whether this phosphatase domain is important for effector export in *Toxoplasma* is not yet known. Given that the kinase domain of ROP17 is necessary for GRA16 export (12), it is intriguing that two of the eight factors necessary for effector export are either a kinase or a putative phosphatase. There are numerous serine residues that are phosphorylated among MYR1, MYR2, MYR3, and MYR4, supporting the possibility that phosphorylation of the translocation machinery is critical to regulating its function in effector export. Phosphoproteomic analysis of these MYR components in parasites lacking GRA44 (a predicted phosphatase) and ROP17 (a protein kinase) may provide insights into the mechanism and regulation of effector export and the interplay of these proteins.

While this work was in progress, we learned of similar studies by Blakely et al., who also found that GRA44 associates with MYR1 and is necessary for efficient c-Myc upregulation during infection (40). The latter authors used a knockdown approach to study GRA44 and saw a more dramatic impact of GRA44 loss on parasite growth than we and others (30) observed for the GRA44 knockout; this might indicate that compensatory changes were selected for during the prolonged selection necessary to generate and expand our knockout clone, as was reported for AMA1 knockouts, which showed a dramatic upregulation of the paralogue, AMA2 (41). Interestingly, GRA44 has a paralog, TGGT1_228160, that is expressed in the *Toxoplasma* sexual stages (42). While the growth phenotype is apparently rescued to a degree sufficient to allow our GRA44 knockout mutant to survive, the effector export phenotype appeared to remain fully impaired, suggesting that whatever potential compensatory changes occurred, they did not restore effector export to the host nucleus. Thus, further analysis of the GRA44 knockout mutant and the potential compensations that it has experienced may reveal clues to other specific functions of GRA44 in *Toxoplasma* tachyzoites.

Our results highlight the question of why some parasite proteins are proteolytically processed by ASP5 and why ASP5 is essential for effector translocation across the PVM. MYR1, GRA44, and GRA45 all possess RRL motifs that appear to be cleaved in an ASP5-dependent manner, yet, surprisingly, their function in the export of GRA16 and of GRA24, in the case of MYR1 (24), appears agnostic to mutation of these sites. For MYR1, we previously showed that the two domains generated by ASP5 processing stay connected through a disulfide bond after cleavage (11); it remains to be determined whether the polypeptides formed by RRL cleavage in GRA44 and GRA45 likewise associate in a similar manner. It is also important to note that our assays may not have been sensitive enough to detect small changes in protein abundance in the host nucleus and that it is the combination of multiple proteins not being processed by ASP5 rather than the result of failure to cleave any single protein that is deleterious to export in Δ*asp5* mutants.

Interestingly, there were a large number of proteins that were more highly enriched than MYR3 in our immunoprecipitations with MYR1, and it remains a strong possibility that additional MYR1-associating proteins are involved in effector translocation. Due to the large number of enriched proteins and the limited throughput of our approach, we were unable to investigate all candidates for such a role; nevertheless, our data showing that GRA16-HA export is not lost in parasites disrupted in *GRA7*, *CST1*, *MAF1*, *PPM3C*, or *GRA54* strongly suggest that it is not general PV/PVM disruption that results in the loss of effector translocation. Further work will be needed to determine which of the remaining proteins that we see enriched in the MYR1 immunoprecipitations are there because of a specific association with MYR1 versus because of nonspecific associations of proteins within the PV/PVM due to an association within lipid rafts or other entities.

Lastly, none of *GRA44*, *GRA45*, or *MYR4* was identified in the forward genetic screen of parasites that are unable to induce c-Myc (10). This could be due to the growth defects observed in *Δmyr4*, *Δgra44*, and Δ*gra45* parasites shown here, since parasites with null mutations in these genes might be lost during the 5 to 7 rounds of selection used in that screen due to a fitness disadvantage. Alternatively, the mutagenesis-based genetic screen was not saturating, and so a more comprehensive, genome-wide screen using CRISPR/Cas9 technologies might reveal these and other genes responsible for effector translocation in *Toxoplasma*. Regardless, our finding of three new components of the export machinery provides a richer understanding of how *Toxoplasma* delivers effectors into host cells. Future work will determine the precise function of each, including how they interact, the role of ASP5 cleavage, and which, if any, constitutes the actual translocon.

## MATERIALS AND METHODS

### Parasite strains, culture, and infections.

All *Toxoplasma* tachyzoites used in this study are in the type I RH strain background, either RH::*MYR1-3×HA* (11), RHΔ*gra16::GRA16-HA* (6), RHΔ*maf1* (33), RHΔ*hpt* (43), or RHΔ*hpt*Δ*ku80* (44). These tachyzoites and all subsequently generated lines were propagated in human foreskin fibroblasts (HFFs) cultured in complete Dulbecco’s modified Eagle medium (DMEM) supplemented with 10% heat-inactivated fetal bovine serum (FBS; HyClone, Logan, UT), 2 mM l-glutamine, 100 U/ml penicillin, and 100 μg/ml streptomycin at 37°C with 5% CO_2_. The HFFs were obtained from the neonatal clinic at Stanford University following routine circumcisions that are performed at the request of the parents for cultural, health, or other personal medical reasons (i.e., not in any way related to research). These foreskins, which would otherwise be discarded, are fully deidentified and therefore do not constitute human subjects research.

Prior to infection, parasites were scraped and syringe lysed using a 27-gauge needle, counted using a hemocytometer, and added to the HFFs. Mock infection was done by first syringe lysing uninfected HFFs, processing these in the same manner used for the infected cells, and then adding the same volume of the resulting material used for infections. For experiments where the human c-Myc protein was detected, the parasites were added to HFFs in medium containing 0% serum.

### Immunofluorescence assay (IFA).

Infected cells grown on glass coverslips were fixed and permeabilized using 100% cold methanol for 10 min. Samples were washed 3 times with phosphate-buffered saline (PBS) and blocked using 3% bovine serum albumin (BSA) in PBS for 1 h at room temperature (RT). HA was detected with rat monoclonal anti-HA antibody 3F10 (Roche), SAG1 was detected with mouse anti-SAG1 monoclonal antibody DG52 (45), GRA7 was detected with rabbit anti-GRA7 antibodies (46), V5 was detected with a mouse anti-V5 tag monoclonal antibody (Invitrogen), and c-Myc was detected with rabbit monoclonal anti-c-Myc antibody Y69 (Abcam). Primary antibodies were detected with goat polyclonal Alexa Fluor-conjugated secondary antibodies (Invitrogen). Both the primary and secondary antibodies were diluted in 3% BSA in PBS. Coverslips were incubated with the primary antibodies for 1 h at RT, washed, and incubated with secondary antibodies for 1 h at RT. Vectashield with DAPI (4′,6-diamidino-2-phenylindole) stain (Vector Laboratories) was used to mount the coverslips on slides. Fluorescence was detected using wide-field epifluorescence microscopy, and images were analyzed using ImageJ software. All images shown for any given condition/staining in any given comparison/data set were obtained using identical parameters.

### Transfections.

All transfections were performed using the Amaxa 4D Nucleofector (Lonza) model. Tachyzoites were mechanically released in PBS, pelleted, and resuspended in 20 μl P3 primary cell Nucleofector solution (Lonza) with 5 to 25 μg DNA for transfection. After transfection, parasites were allowed to infect HFFs in DMEM.

### Plasmid construction.

For gene disruption plasmids, guide RNAs, designed against a PAM site of each gene of interest, were cloned into the pU6-Universal plasmid. pU6-Universal was a gift from Sebastian Lourido (Addgene plasmid number 52694; http://n2t.net/addgene:52694; RRID:Addgene_52694).

For ectopic expression plasmids, the pGRA-V5 plasmid was created by replacing the HA tag sequence in the pGRA-HPT-HA plasmid (47) with the V5 tag DNA sequence (GGCAAGCCCATCCCCAACCCCCTGCTGGGCCTGGACAGCAC) and removing the hypoxanthine-xanthine-guanine phosphoribosyltransferase (HPT) resistance cassette using standard molecular biology techniques. The pX-V5 plasmid was created by removing the GRA1 promoter from pGRA-V5 using standard molecular biology techniques. Complementation plasmids to ectopically express V5-tagged proteins off their native promoters were created by PCR amplification of the open reading frame of each gene, minus the stop codon, plus ∼2,000 bp upstream of the start codon to include the native promoter, followed by cloning into pX-V5 using Gibson Assembly (New England Biolabs [NEB]). RRL → ARL- or RRL → AAA-mutated complementation plasmids were generated using overlap extension PCR and primers harboring the mutation and cloning the resultant products into pX-V5 using Gibson Assembly (NEB).

For endogenous tagging plasmids, approximately 1,500 to 3,000 bp of the 3′ coding sequence of each gene was amplified from RH genomic DNA and cloned into the pTKO2-HPT-3×HA plasmid (11) either by using Gibson Assembly (NEB) or by cloning into the EcoRV and NotI restriction sites.

A list of all primers and plasmids used and generated in this study can be found in Data Set S2 in the supplemental material.

10.1128/mSphere.00858-19.8 DATA SET S2 Primers, sgRNA sequences, and plasmids used and/or generated in this study. Download Data Set S2, XLSX file, 0.01 MB. Copyright © 2020 Cygan et al. 2020 Cygan et al. This content is distributed under the terms of the Creative Commons Attribution 4.0 International license.

### Endogenous tagging.

Endogenous tagging plasmids were transfected into *Toxoplasma* via electroporation. Tachyzoites were allowed to infect HFFs in T25 flasks for 24 h, after which the medium was changed to complete DMEM supplemented with 50 μg/ml mycophenolic acid and 50 μg/ml xanthine for selection for the hypoxanthine-xanthine-guanine-phosphoribosyltransferase (HXGPRT or HPT) marker for 3 to 5 days.

### Gene disruption.

A list of all sgRNA sequences used in this study can be found in Data Set S2. RHΔ*gra16*::*GRA16-HA* tachyzoites were transfected with pTKO2-CAT-mCherry (CAT is chloramphenicol acetyltransferase, which confers resistance to chloramphenicol; the plasmid was a gift from Ian Foe and Matthew Bogyo [48]) and the corresponding modified pU6-sgRNA plasmid and allowed to infect HFFs for 24 to 48 h. For gene disruption of MYR3, the previously published pSAG1:U6-Cas9:sgMYR3 plasmid was used instead (11). At between 24 and 48 h after transfection, DMEM with 80 μM chloramphenicol was added to the cells. The medium was replaced with fresh chloramphenicol-supplemented medium every 48 to 72 h. After at least 7 days in selection, single clones were selected from the transfected populations in 96-well plates using limiting dilution. Single clones were maintained in chloramphenicol-supplemented medium until confirmation of the genetic disruption.

### Ectopic expression.

Plasmids for ectopic expression were transiently transfected into *Toxoplasma* using electroporation. Tachyzoites were allowed to infect HFFs for 18 to 24 h before assessing for expression of the ectopically expressed protein via either IFA or Western blotting.

### Western blotting.

Cell lysates were prepared at the time points postinfection indicated above in Laemmli sample buffer (Bio-Rad). The samples were boiled for 5 min, separated by SDS-PAGE, and transferred to polyvinylidene difluoride (PVDF) membranes. The membranes were blocked with 5% nonfat dry milk in Tris-buffered saline supplemented with 0.5% Tween 20, and proteins were detected by incubation with primary antibodies diluted in blocking buffer, followed by incubation with secondary antibodies (raised in goat against the appropriate species) conjugated to horseradish peroxidase (HRP) and diluted in blocking buffer. HA was detected using an HRP-conjugated HA antibody (catalog no. 12013819001; Roche), SAG2A was detected using rabbit polyclonal anti-SAG2A antibodies (49), cyclin E1 was detected using mouse monoclonal antibody HE12 (Santa Cruz Biotechnology), and GAPDH (glyceraldehyde-3-phosphate dehydrogenase) was detected using mouse monoclonal anti-GAPDH antibody 6C5 (Calbiochem). Horseradish peroxidase (HRP) was detected using an enhanced chemiluminescence (ECL) kit (Pierce).

### Plaque assay.

Parasites were syringe released from HFFs and added to confluent HFFs in T25 flasks. After 7 days, the infected monolayers were washed with PBS, fixed with methanol, and stained with crystal violet. The plaque area was measured using ImageJ software.

### IPs for mass spectrometry.

Immunoprecipitations (IPs) to identify MYR1-interacting proteins in HFFs were performed as follows. Cells in one 15-cm dish of HFFs for each infection condition were grown to confluence. HFFs were infected with either 15 × 10^6^ RH::*MYR1-3×HA* or RHΔ*hpt* parasites for 24 h. Infected cells were washed 3 times in cold PBS and then scraped into 1 ml cold cell lysis buffer (50 mM Tris [pH 8.0], 150 mM NaCl, 0.1% [vol/vol] Nonidet P-40 alternative [CAS no. 9016-45-9]) supplemented with complete protease inhibitor cocktail (cOmplete, EDTA free; Roche). Cell lysates were passed 3 times through a 25-gauge needle, followed by passage 3 times through a 27-gauge needle and sonication on ice (Branson Sonifier 250), consisting of 3 pulses of 10 s each at a 50% duty cycle and output control 2. The cell lysates were spun at 1,000 × *g* for 10 min to remove insoluble material and unlysed cells. Lysates were added to 100 μl magnetic beads conjugated to anti-HA antibodies (Pierce), and the mixture was incubated overnight with rotation at 4°C. Unbound protein lysate was removed, and the anti-HA magnetic beads were then washed 10 times in cell lysis buffer. HA-tagged MYR1 and associated proteins were eluted in 60 μl pH 2.0 buffer (Pierce) for 10 min at 50°C to dissociate the proteins from the antibody-conjugated beads. The elutions were immediately neutralized 1:10 with pH 8.5 neutralization buffer (Pierce).

### Mass spectrometry sample preparation.

Forty-five microliters of each IP elution was combined with 15 μl of 4× Laemmli sample buffer supplemented with β-mercaptoethanol (Bio-Rad), boiled for 10 min at 95°C, and loaded on a Bolt 4 to 12% bis-Tris gel (Invitrogen). The samples were resolved for approximately 8 min at 150 V. The gel was washed once in ultrapure water (Thermo) and fixed in 50% methanol and 7% acetic acid for 15 min, followed by 3 additional washes with ultrapure water. The gel was stained for 10 min with the GelCode Blue reagent (Thermo) and washed with ultrapure water for an additional 20 min. One gel band (approximately 1.5 cm in size) for each condition was excised and destained for 2 h in a 50% methanol and 10% acetic acid solution, followed by a 30-min soak in ultrapure water. Each gel slice was cut into 1-mm by 1-mm squares, covered in 1% acetic acid solution, and stored at 4°C until the in-gel digestion could be performed.

To prepare samples for mass spectrometry, the 1% acetic acid solution was removed, 10 μl of 50 mM dithiothreitol (DTT) was added, and the volume was increased to 100 μl with 50 mM ammonium bicarbonate. Samples were incubated at 55°C for 30 min. The samples were then brought down to RT, the DTT solution was removed, 10 μl of 100 mM acrylamide (propionamide) was added, and the volume was again normalized to 100 μl with 50 mM ammonium bicarbonate, followed by an incubation at RT for 30 min. Acrylamide solution was removed, 10 μl (0.125 μg) of trypsin-LysC (Promega) solution was added, and another 50 μl of 50 mM ammonium bicarbonate was added to cover the gel pieces. Samples were incubated overnight at 37°C for peptide digestion. The solution consisting of digested peptides was collected in fresh Eppendorf tubes, 50 μl of extraction buffer (70% acetonitrile, 29% water, 1% formic acid) was added to the gel pieces, and the mixture was incubated at 37°C for 10 min, centrifuged at 10,000 × *g* for 2 min, and collected in the same tubes consisting of the previous eluate. This extraction was repeated one more time. Collected extracted peptides were dried to completion in a SpeedVac apparatus and stored at 4°C until they were used for mass spectrometry.

### Mass spectrometry.

Eluted, dried peptides were dissolved in 12.5 μl of 2% acetonitrile and 0.1% formic acid, and 3 μl was injected into an in-house-packed C_18_ reversed-phase analytical column (15 cm in length). Peptides were separated using a Waters M-class ultraperformance liquid chromatograph, operated at 450 nl/min, using a linear 80-min gradient from 4 to 40% mobile phase B. Mobile phase A consisted of 0.2% formic acid, 99.8% water; mobile phase B was 0.2% formic acid, 99.8% acetonitrile. Ions were detected using an Orbitrap Fusion mass spectrometer operating in a data-dependent fashion using typical top-speed methodologies. Ions were selected for fragmentation based on the most intense multiply charged precursor ions using collision-induced dissociation (CID). Data from these analyses were then transferred for analysis.

### Mass spectrometric analysis.

The .RAW data were searched using the MaxQuant (v1.6.1.0) tool against the canonical human database from UniProt, *Toxoplasma* GT1 databases from ToxoDB (v7.3 and v37.0), and the built-in contaminant database. Specific parameters used in the MaxQuant analysis can be found in Data Set S1. Peptide and protein identifications were filtered to a 1% false discovery rate (FDR), and reversed proteins, contaminants, and proteins identified by only a single modification site were removed from the data set. MYR1-3×HA enrichment over the non-HA-tagged RH was determined by adding 1 to each spectral count (tandem MS [MS/MS] count) and calculating the NSAF (the number of spectral counts identifying a protein divided by the protein’s length divided by the sum of all spectral counts/lengths for all proteins in the experiment). The average MYR1-3×HA enrichment from the two biological replicates (IP 1 and IP 2) was used to determine the protein ranking.

### Data availability.

The mass spectrometry proteomics data have been deposited in the ProteomeXchange Consortium (http://proteomecentral.proteomexchange.org) via the PRIDE partner repository (50) with the data set identifier PXD016383.
