# Supplementary figures and images for: Coimmunoprecipitation with MYR1 Identifies Three Additional Proteins within the Toxoplasma gondii Parasitophorous Vacuole Required for Translocation of Dense Granule Effectors into Host Cells
Source: mSphere. 2020 Feb 19;5(1):e00858-19. doi: 10.1128/mSphere.00858-19 (PMC7031616; doi:10.1128/mSphere.00858-19)

Supplemental Figure 1.

**A**

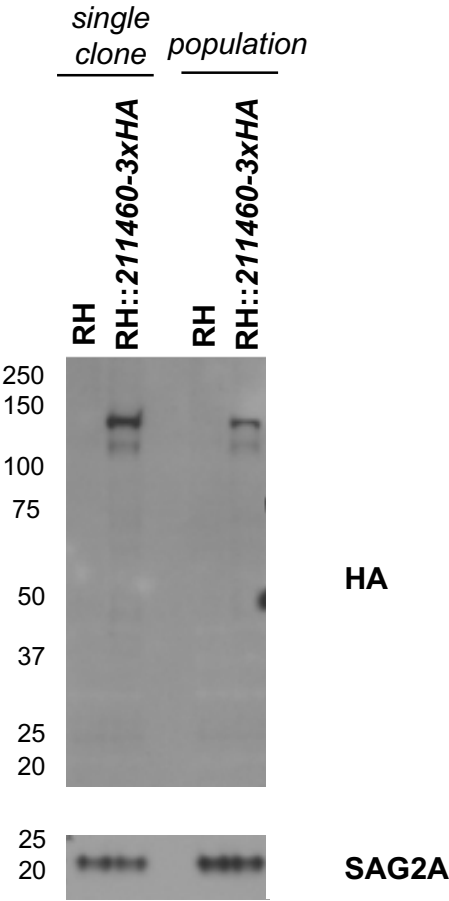

**B**

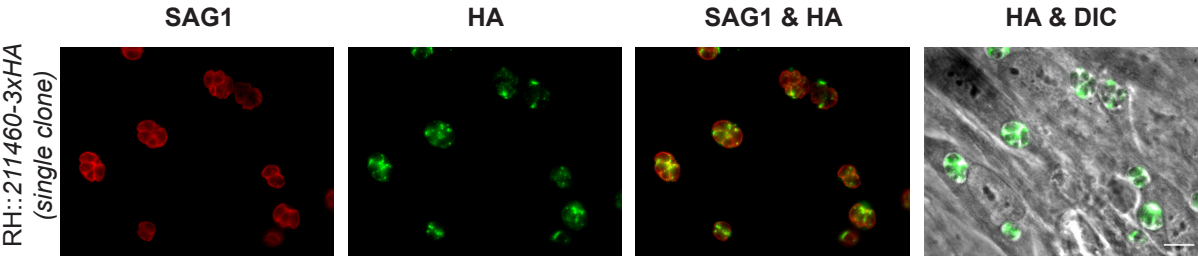

Supplement: FIG S1 [file mSphere.00858-19-sf001.pdf]

Supplemental Figure 2.

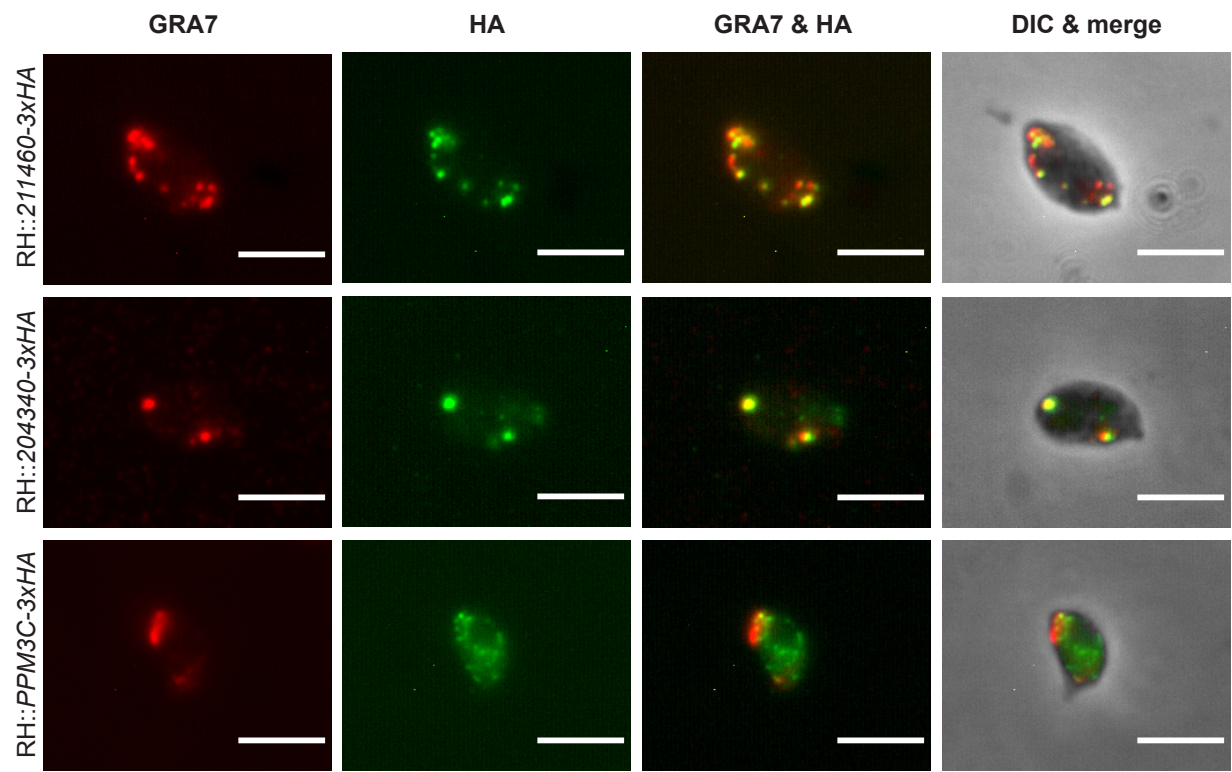

Supplement: FIG S2 [file mSphere.00858-19-sf002.pdf]

Supplemental Figure 3.

**A**

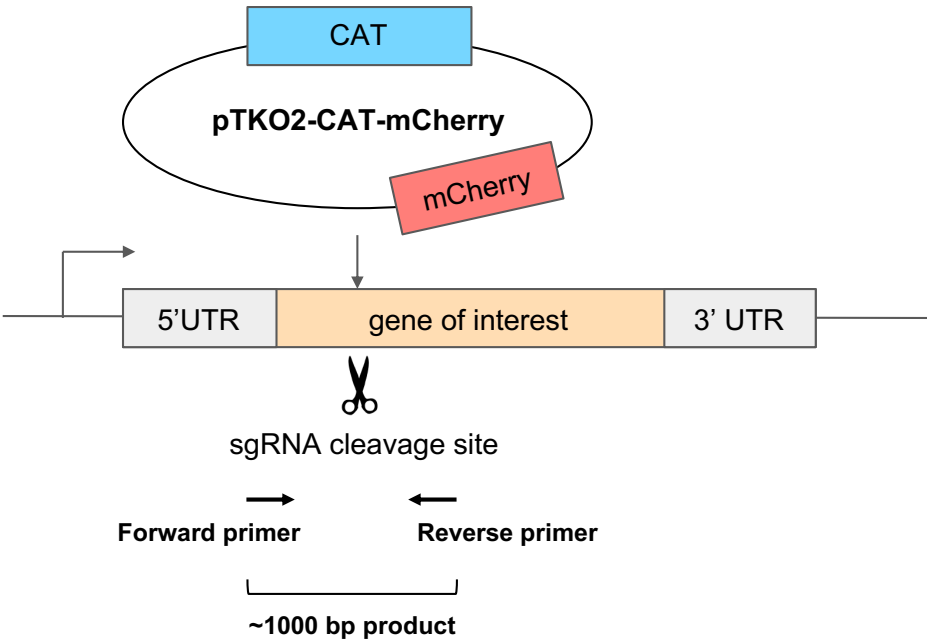

**B**

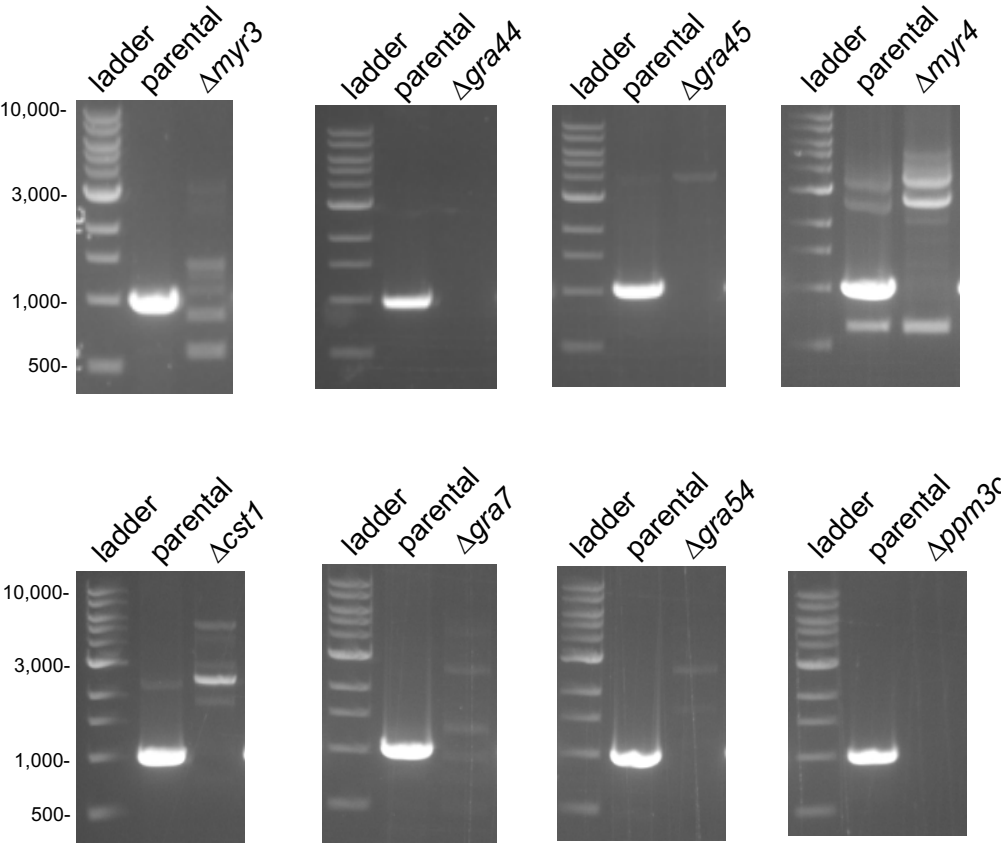

Supplement: FIG S3 [file mSphere.00858-19-sf003.pdf]

Supplemental Figure 4.

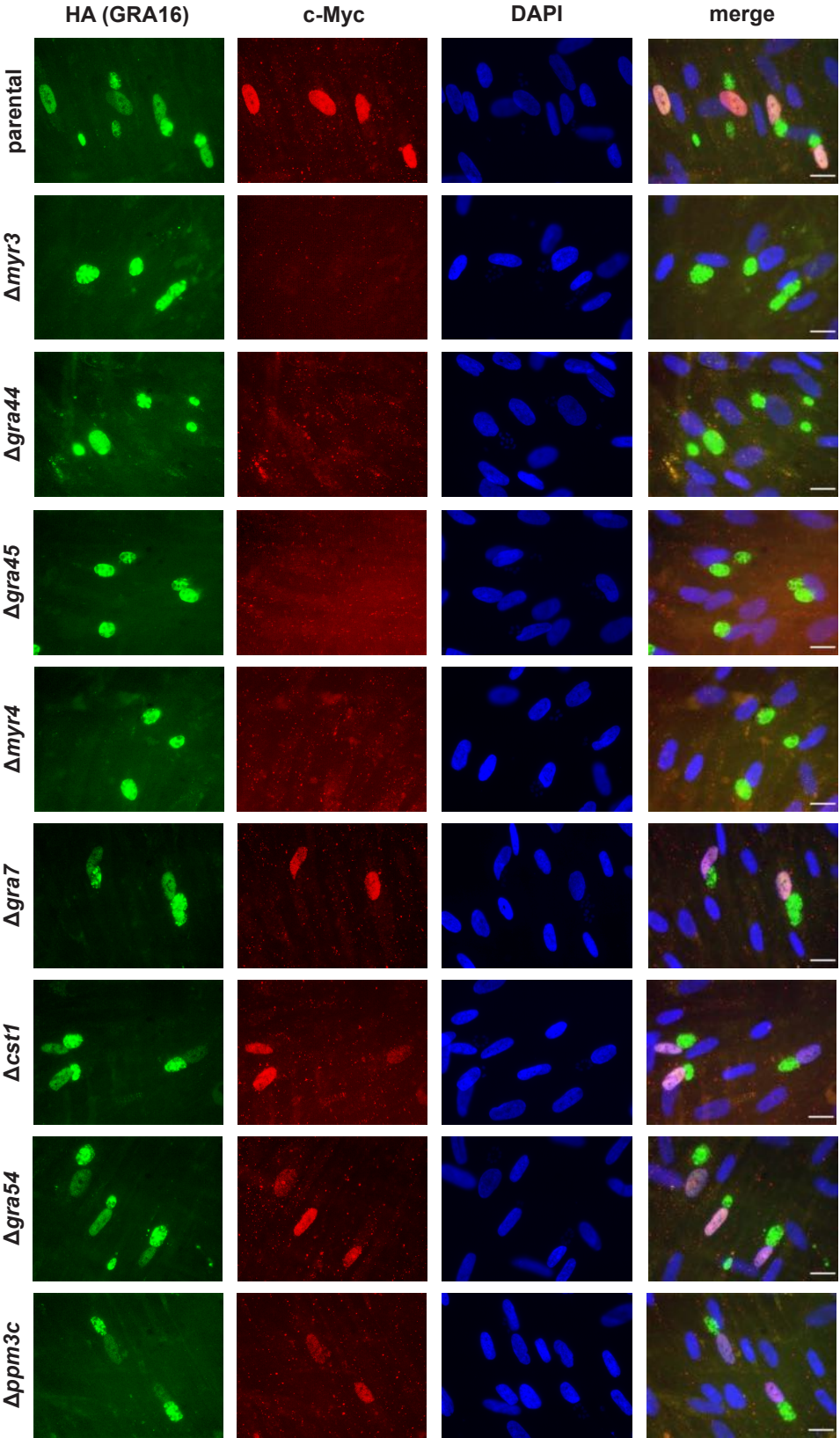

Supplement: FIG S4 [file mSphere.00858-19-sf004.pdf]

Supplemental Figure 5.

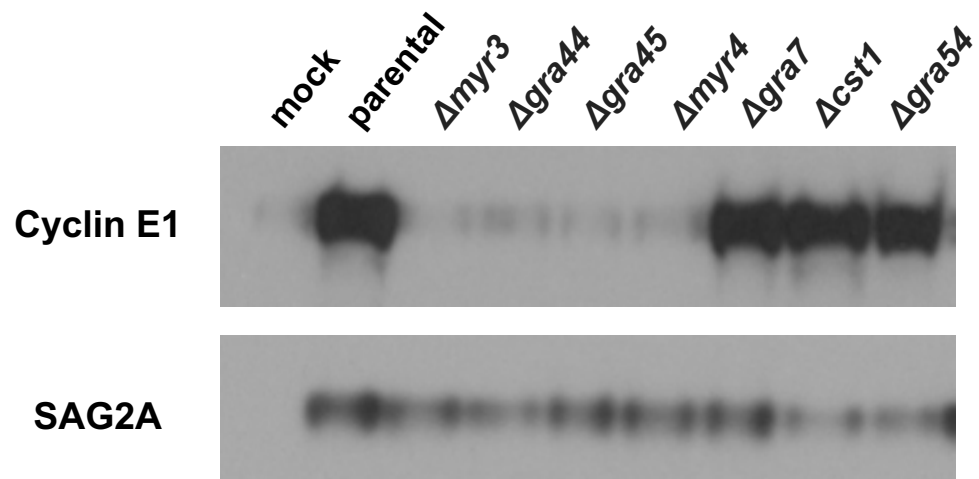

Supplement: FIG S5 [file mSphere.00858-19-sf005.pdf]
